# Finite element analysis of double‐plate fixation using reversed locking compression‐distal femoral plates for Vancouver B1 periprosthetic femoral fractures

**DOI:** 10.1186/s12891-021-04152-5

**Published:** 2021-03-13

**Authors:** Daisuke Takahashi, Yoshihiro Noyama, Tsuyoshi Asano, Tomohiro Shimizu, Tohru Irie, Mohamad Alaa Terkawi, Norimasa Iwasaki

**Affiliations:** 1 Department of Orthopaedic Surgery, Faculty of Medicine, Graduate School of Medicine, Hokkaido University, Kita-15, Nishi-7, Kita-ku, Sapporo, Japan; 2 Department of Pharmaceutical Affairs Division, Teijin Nakashima Medical Company Limited, 688-1, Joto-Kitagata, Higashi-ku, Okayama, Japan

**Keywords:** Periprosthetic femoral fracture, Double plate, Vancouver B1, Finite element analysis, Locking compression‐distal femoral plate

## Abstract

**Background:**

Internal fixation is recommended for treating Vancouver B1 periprosthetic femoral fractures. Although several fixation procedures have been developed with high fixation stability and union rates, long-term weight-bearing constructs are still lacking. Therefore, the aim of the present study was to evaluate the stability of a double-plate procedure using reversed contralateral locking compression-distal femoral plates for fixation of Vancouver B1 periprosthetic femoral fractures under full weight-bearing.

**Methods:**

Single- and double-plate fixation procedures for locking compression-distal femoral plates were analysed under an axial load of 1,500 N by finite element analysis and biomechanical loading tests. A vertical loading test was performed to the prosthetic head, and the displacements and strains were calculated based on load-displacement and load-strain curves generated by the static compression tests.

**Results:**

The finite element analysis revealed that double-plate fixation significantly reduced stress concentration at the lateral plate place on the fracture site. Under full weight-bearing, the maximum von Mises stress in the lateral plate was 268 MPa. On the other hand, the maximum stress in the single-plating method occurred at the defect level of the femur with a maximum stress value of 1,303 MPa. The principal strains of single- and double-plate fixation were 0.63 % and 0.058 %, respectively. Consistently, in the axial loading test, the strain values at a 1,500 N loading of the single- and double-plate fixation methods were 1,274.60 ± 11.53 and 317.33 ± 8.03 (× 10^− 6^), respectively.

**Conclusions:**

The present study suggests that dual-plate fixation with reversed locking compression-distal femoral plates may be an excellent treatment procedure for patients with Vancouver B1 fractures, allowing for full weight-bearing in the early postoperative period.

## Background

Total hip arthroplasty (THA) is a surgical procedure for hip joint replacement with an artificial prosthesis that has been proven to improve quality of life for the majority of patients with hip disability. Periprosthetic femoral fractures are serious complications of THA that often require revision surgery [1–3]. Treatment decisions for periprosthetic femoral fractures are typically made based on the Vancouver classification system [4, 5]. Vancouver type B1 femoral fractures occur around the stem tip with a stable implant and are often associated with complications characterised by non-union and implant failure [2, 6, 7].

Internal fixation is recommended as the treatment to minimise the risk of prosthetic loosening and reduce early mobilisation [8–10]. There are various procedures for internal fixation, most of which have shown good clinical outcomes. Recent biomechanical studies demonstrated that locking plate constructs results in greater stiffness than conventional cable plating [11–14]. Nonetheless, single locking plate fixation may not always offer optimal fixation, and failure or less satisfactory results have often been reported. Such cases most likely occur as a result of full weight-bearing on the plate system [15, 16]. Additional attachment of an anterior plate is suggested to improve fracture stability and has shown some satisfactory results in biomechanical studies [17, 18]. In these studies, however, the lateral locking plates were too short and narrow, which may limit their clinical applications. An alternative approach of using a reversed contralateral distal femoral locking plate has been reported for the treatment of Vancouver B1 fractures, with a good fracture union rate. However, there is always a risk of failure due to plate weight-bearing [19–21]. Addition of an anterior locking plate to the reversed contralateral locking compression-distal femoral plates (LCP-DF) might be a good choice to improve fixation stability and overcome weight-bearing restrictions when using a single-plate system. Therefore, the aim of the current study was to evaluate the potential advantages of a reversed contralateral LCP-DF double-plate fixation procedure for treatment of Vancouver B1 fractures under full weight-bearing using finite element analysis (FEA) and biomechanical testing.

## Materials and Methods

### Construction of the finite element analysis model

**Fig. 1:**
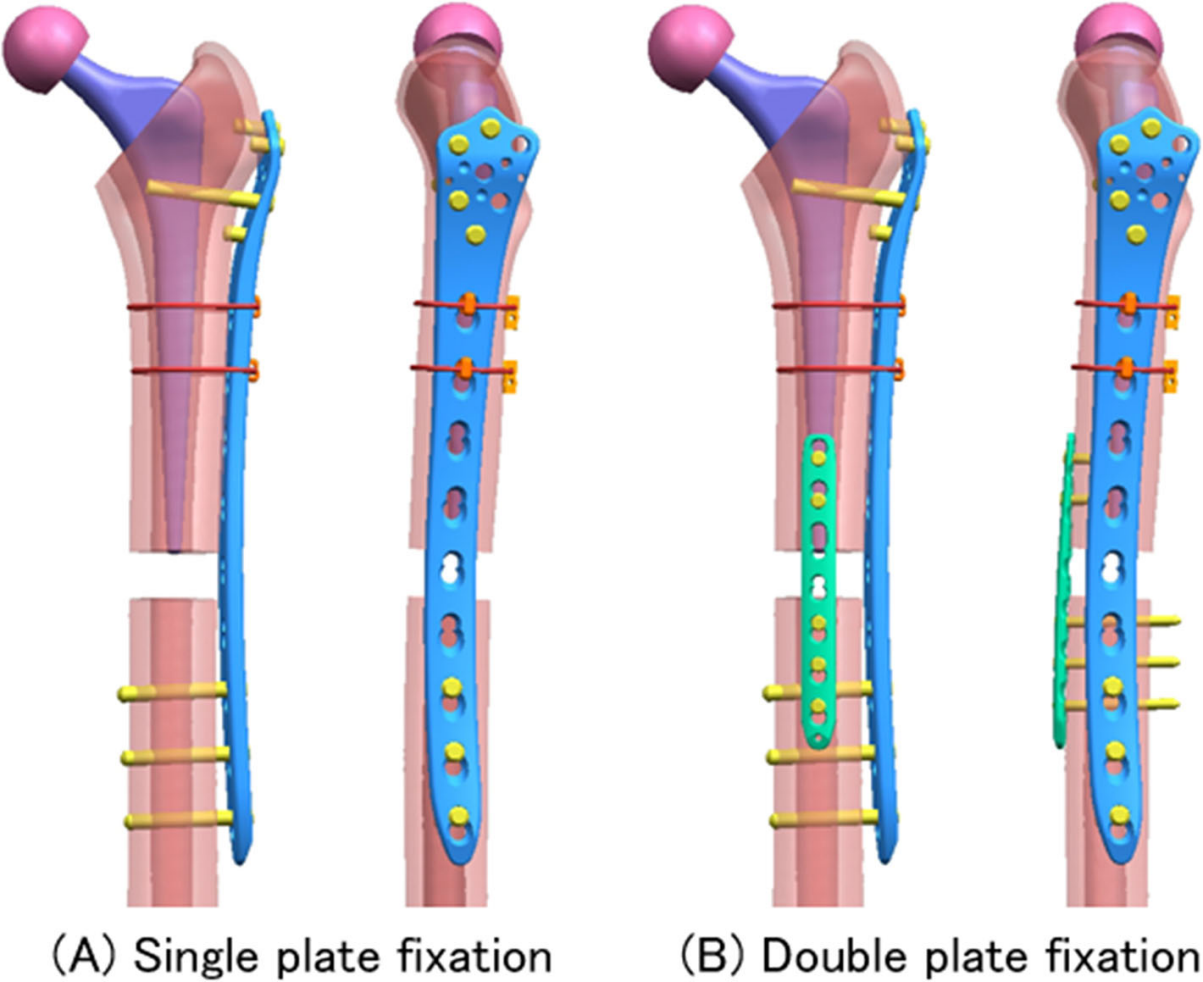
3D model of two different fixation methods for finite element analysis We created the figure using the 3D-CAD software UG NX 5(SIEMENS)

A three-dimensional (3D) model of composite femurs (4th generation, Sawbones Worldwide, WA) was constructed by computed tomography (CT) imaging (Mimics 16, Materialise, Software & Services for Biomedical Engineering, Leuven, Belgium) of the data obtained from CT (Eclos-4 S, Hitachi, Otawara, Tochigi, Japan) [22]. The periprosthetic femoral fracture model was assembled in a 3D-computer aided design software (UG NX 5, SIEMENS, Plano, TX), and the stem position was determined based on radiographs and CT data of an experimental THA model. A transverse fracture was created 10 mm below the tip of the Exeter femoral stem (Stryker, Kalamazoo, MI) and the construct was fixed using two different fixation methods (Fig. 1). The single-plate method was performed by fixing a 9-hole LCP-DF locking plate (Depuy Synthes, West Chester, PA) laterally with four proximal uni-cortical locking screws and three distal bi-cortical locking screws. The double-plate method was performed similarly to the single-plating method with an additional anterior 7-hole metaphyseal locking plate (Depuy Synthes) with two proximal uni-cortical locking screws and three distal bi-cortical locking screws. To provide additional fixation to the proximal fragments, two cerclage cables (Depuy Synthes) were used with a tension of 400 N.

### Material properties

All sections were assigned isotropic material properties with an elastic modulus of 16.3 GPa for cortical bone [23], 0.15 GPa for cancellous bone [24], 2.8 GPa for polymethylmethacrylate (PMMA) cement [25], 195 GPa for Orthinox stainless steel [26], and 110 GPa for Titanium [27]. A Poisson’s ratio of 0.3 was used for all materials [26].

### Finite element analysis modelling

**Fig. 2:**
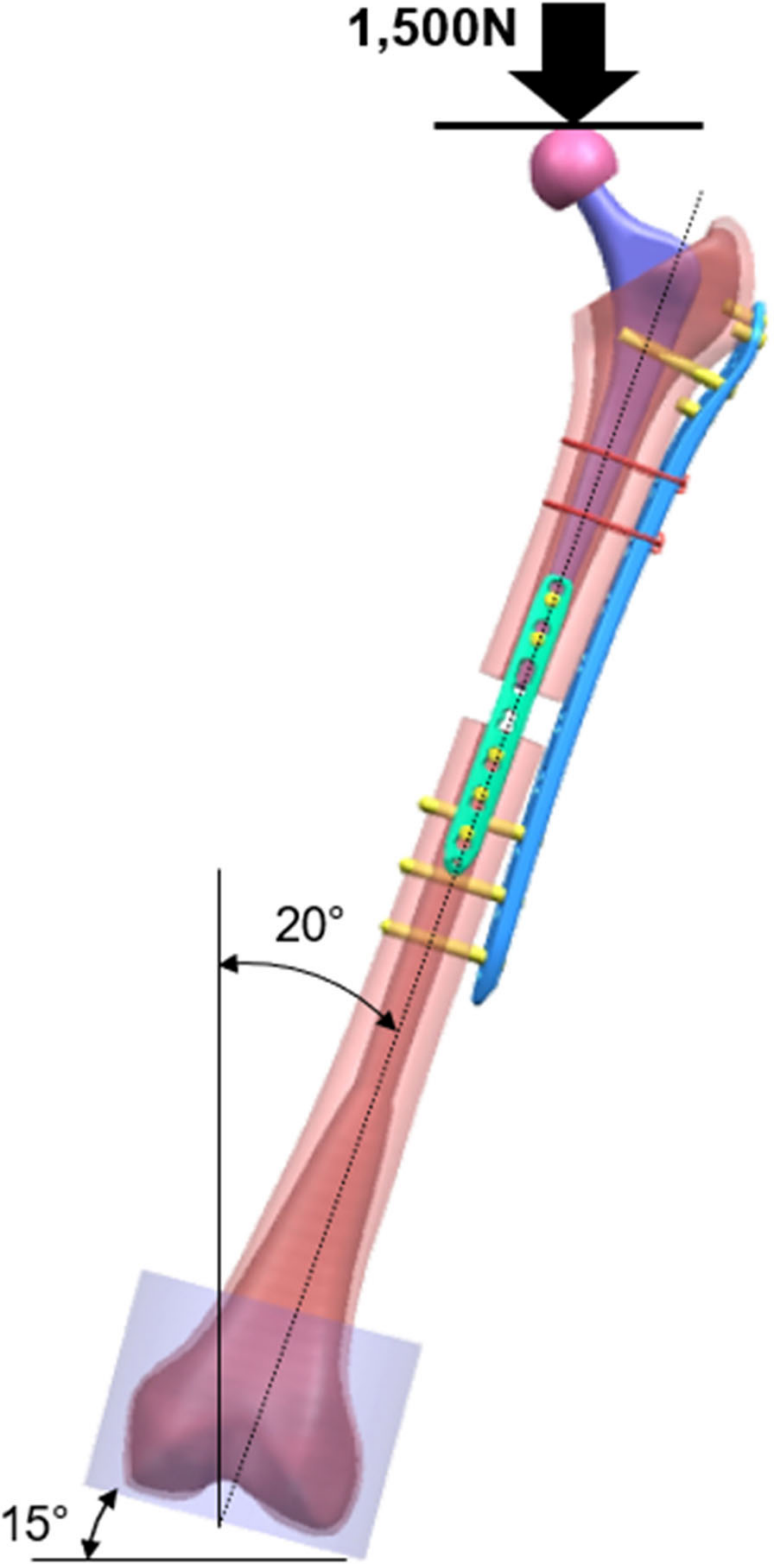
Finite element analysis conditions. The constructs are positioned at 20 degrees of frontal plane adduction and aligned vertically in the sagittal plane. Two different models were analysed under an axial load of 1,500 N. We created the figure using the 3D-CAD software UG NX 5(SIEMENS)

A finite element pre-processor was generated using HyperMesh 13 (Altair Engineering, Troy, MI). Tetrahedral primary elements were used, whereas the number of elements and nodes were 1,023,382 and 224,630 in the single-plate fixation method, and 1,047,309 and 231,601 in the double-plate fixation method, respectively. To set up the boundary conditions, the cortical and trabecular bones were fixed by glue, with a coefficient of friction of 0.1, 0.1, 0.3, 0.1 and 0.1 used at the bone-stem, bone-plate, bone-screw, bone-cable, and cable-fastener interfaces, respectively [28]. The distal end of the femoral model was fixed with cement. These constructs were positioned at 20 degrees of frontal plane adduction and aligned vertically in the sagittal plane. This position was to simulate the anatomical one-legged stance. Thereafter, the constructs were tested under an axial load of 1,500 N (Fig. 2) as previously described [29, 30], and the results were then analysed using a nonlinear FEA software (MSC Marc 2017, MSC Software, Newport Beach, CA).

### Testing and analysis

**Fig. 3:**
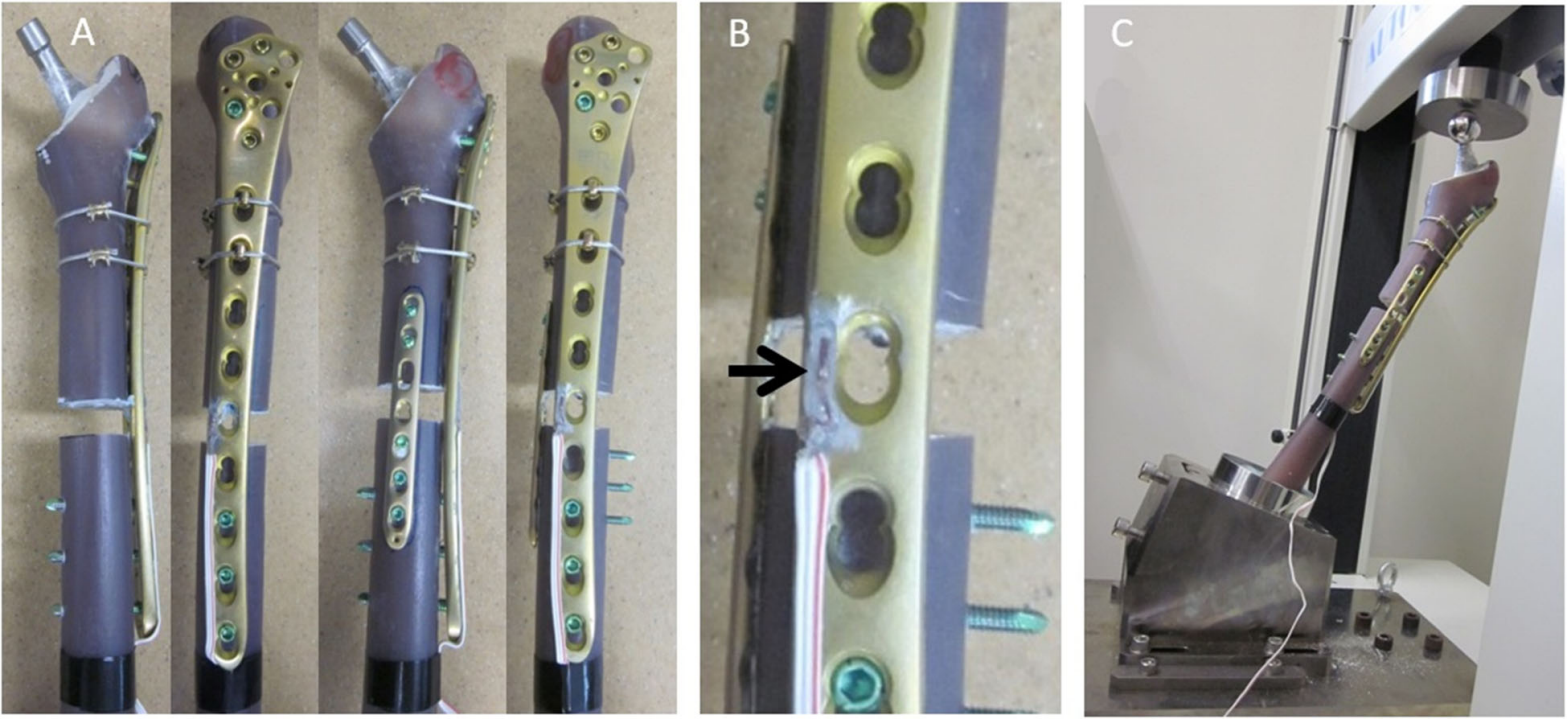
Biomechanical testing apparatus. **a** Single- and double-plate fixation. **b** The strain gauge is attached to the surface of the LCP-DF (arrow). **c** The setup with the axial loading testing

Biomechanical testing was conducted using synthetic composite femurs (Sawbones Worldwide). Composite bones were placed in a bench-mounted vice grip, and then neck osteotomy, trochanteric reaming, and rasping were performed. Polymethyl methacrylate (PMMA) cement (Simplex P, Stryker) was pressurised into composite bone, and an Exeter hip prosthesis (Stryker) was manually inserted. Stem alignment was checked using X-ray (data not shown). To provide additional fixation to the proximal fragments, two cerclage cables (Depuy Synthes) were used with a tension of 400 N (Fig. 3 A). The strain gauge (KFG-2 N-120-C1, Kyowa, Chofu, Japan) was attached to the surface of the LCP-DF, parallel to the plate axis, and at the defect level (Fig. 3B). The distal end of the composite bone was placed in an 80-mm-wide threaded steel pipe and fixed with two steel bolts for anti-rotation. The constructs were further fixed by pouring the cement into the steel pipe, and the fracture fixation models were made with the mechanical test equipment (AGS-H, Shimadzu, Japan). To achieve maximum vertical load directly on the head of the prosthesis, the mounting platform was placed to facilitate biaxial translation of the specimen (Fig. 3 C). For the axial loading test, a sequentially vertical loading test was performed on the prosthetic head at a velocity of 5 mm/min up to 1,500 N. The test was repeated thrice for each construct. The maximum displacements and strains were calculated based on the load-displacement and load-strain curves generated by the static compression tests.

### Data analysis and statistics

Statistical analysis was performed using Student’s *t*-test to compare the differences between two independent groups, and the results were considered significant when *P* < 0.05. Data are presented as means ± standard error.

## Results

**Fig. 4:**
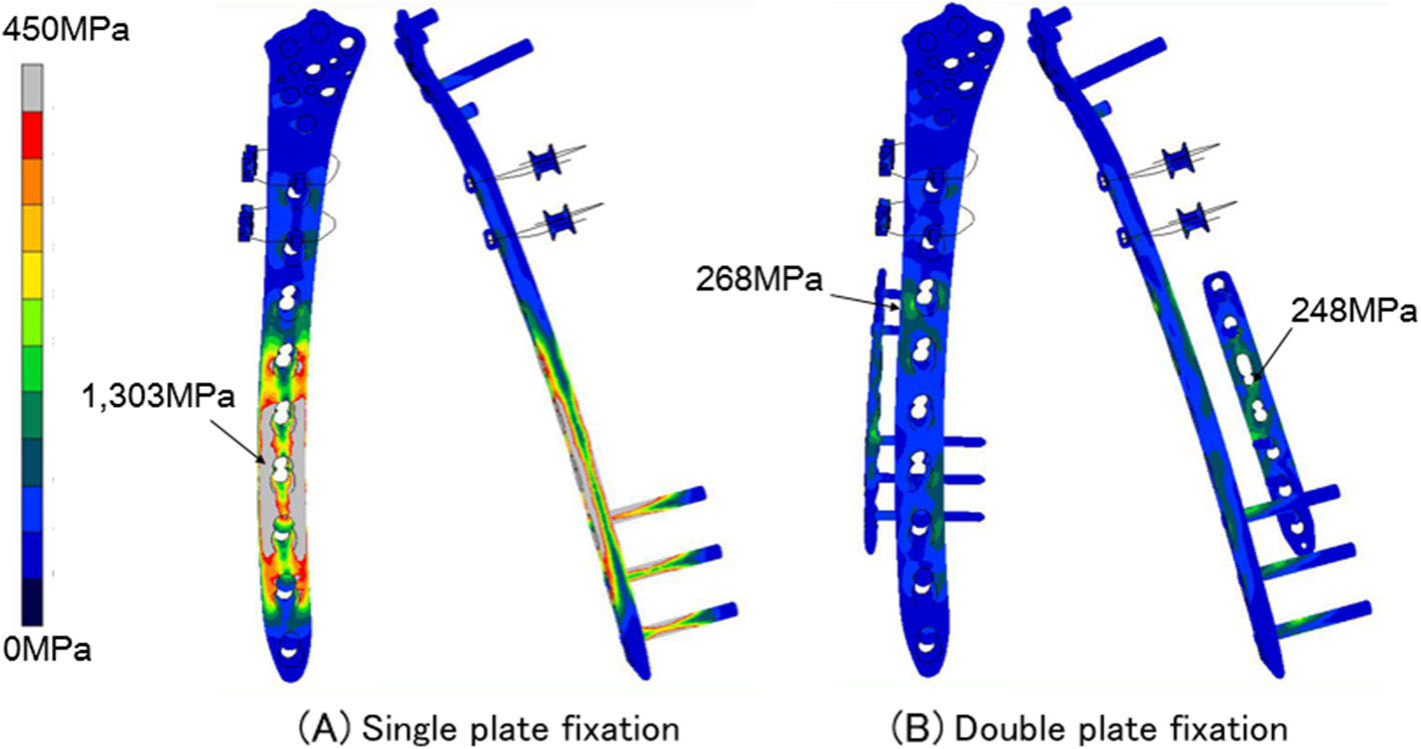
Pattern of von Mises stress distributions of single- and double-plate fixation at 1,500 N of axial loading in finite element analysis

**Fig. 5:**
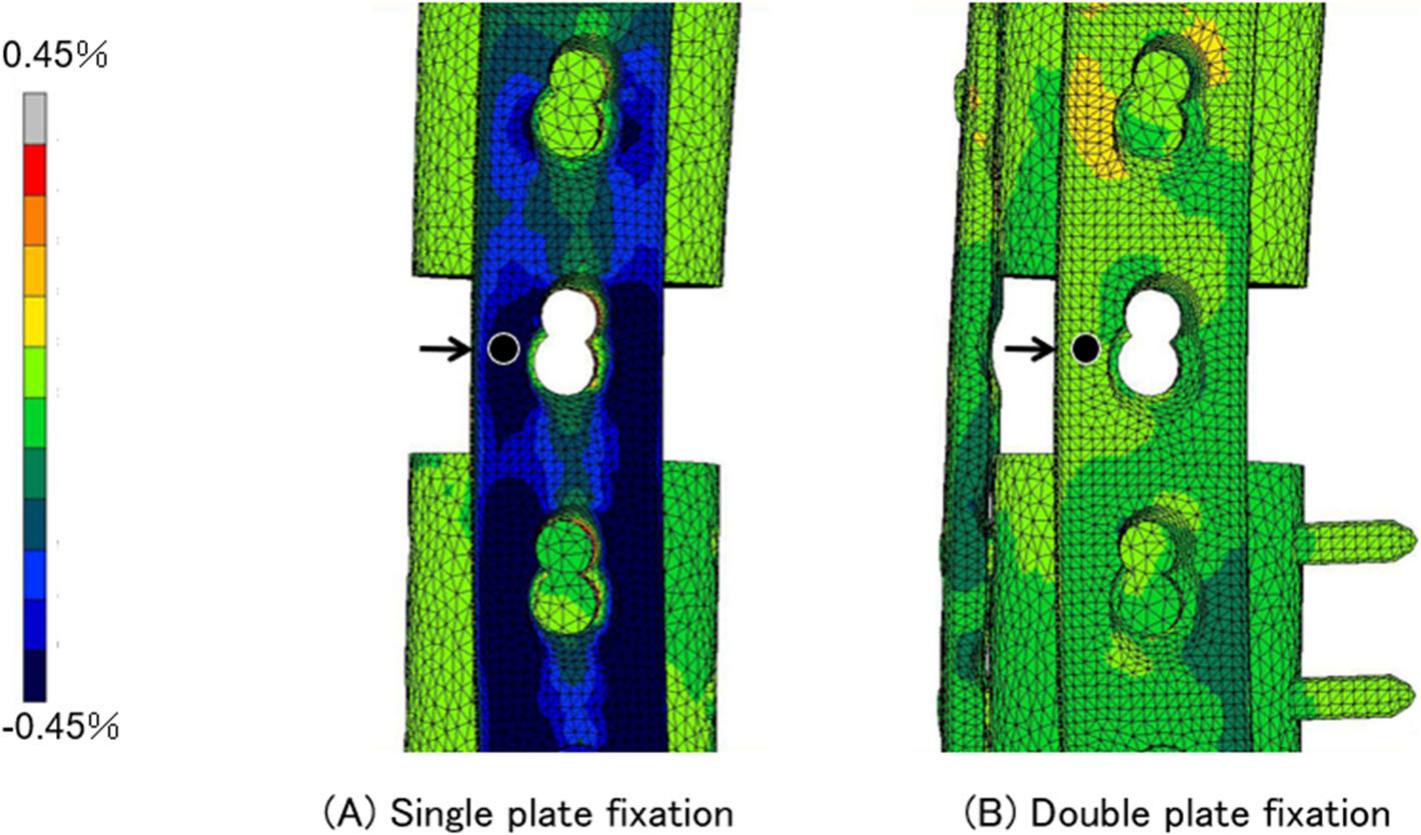
Maximum principal strain distribution of single- and double-plate fixation. Comparison of the maximum principal strain at the anterior side in the lateral LCP-DF plate (arrow)

**Fig. 6:**
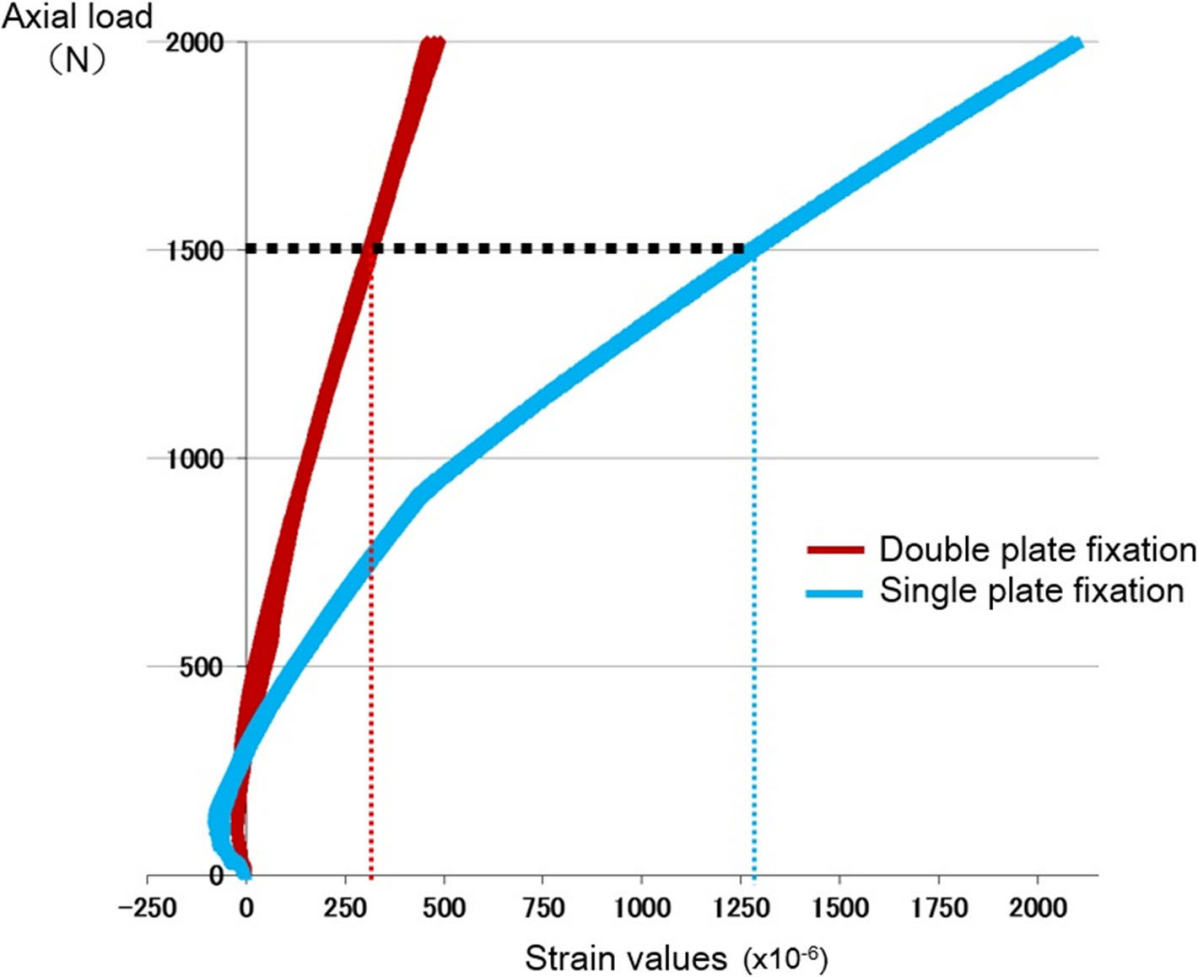
Load-strain curve in axial loading testing. The strain values of double-plate fixation (red line) are significantly lower than those of single-plate fixation (blue line) at a load of 1,500 N (*p* < 0.0001)

To define areas of high stress and stress shielding with single- and double-plate fixation, von Mises stress distributions at 1,500 N of axial loading were determined by FEA (Fig. 4). Of note, the maximum von Mises stress in the single-plating method occurred at the femoral defect level, and the stress areas were present at the centre of the LCP-DF plate, with a maximum stress value of 1,303 MPa. The stress level at the defect level was much lower in the double-plating than in the single-plating method, and the stress level was high at the central part of both plates. The maximum stress value was 268 MPa, located slightly proximal to the centre of the LCP-DF plate and 248 MPa slightly proximal to the centre of the anterior plate (Fig. 4). The maximum principal strains of the single- and double-plate fixation methods at the anterior side of the lateral LCP-DF plate were noted to be distributed parallel to the axis of the plate with values of -0.63 % and 0.058 %, respectively (Fig. 5). To further assess the fixation strength of the plate constructs, biomechanical testing was performed. In the axial loading test, the single-plate fixation strain values were significantly higher than for double-plate fixation above 370 N of axial loading. In addition, the strain values at 1,500 N loading of the single- and double-plate fixation methods were 1,274.60 ± 11.53 and 317.33 ± 8.03 (× 10^− 6^), respectively (Fig. 6). These results suggest that double-plate fixation with lateral LCP-DF offers greater stiffness and stability to the construct than single-plate fixation under full weight-bearing.

## Discussion

Treatment of Vancouver B1 periprosthetic femoral fractures remains one of the major challenges for orthopaedic surgeons. Despite advances in treatment procedures, problems including lack of proximal fixation, non-union, and loss of fixation occur often and require surgical intervention. Locking plates represent the most common choice for fracture treatment and provide stable fracture fixation. Recent biomechanical studies have shown that locking plates have a higher axial loading resistance than conventional cable plate fixation, which is also used to stabilise periprosthetic femoral fractures [11, 31]. However, clinical failure of this treatment has been increasingly reported. For instance, Buttaro et al. [15] reported a high failure rate in the majority of 14 patients with Vancouver B1 fractures treated by single lateral locking plate fixation. This may be due to the extremely high bending forces present at the tip of the prosthesis. On the other hand, the use of distal femoral locking plate fixation leads to successful union in almost all patients, but one patient had delayed union, suggesting a limitation of single-plating procedure [32]. Indeed, a reversed contralateral distal femoral locking plate offers a significant advantage over conventional plating since it allows multiple points of fixation around the trochanteric region of the femur, fitting the anatomical shape of the femur at all levels. Moreover, greater fracture fixation stability was achieved using an additional anterior plate attachment [18]. In a biomechanical study, a lateral plus an anterior locked plate were stiffer than a single-plate fixation method [17]. Considering all these issues, we hypothesised that reversed contralateral LCP-DF plus an additional anterior locked plate would be an appropriate choice for the treatment of Vancouver B1 fractures. Therefore, in the present study, we used contralateral reversed LCP-DF plates as lateral plates to approximate what is done in actual clinical cases and compared single- and double-plate fixation for Vancouver B1 fractures.

Our results showed that the maximum stress level to the lateral plate in the single-plate fixation procedure was higher than the fatigue limits of titanium (ca. 816 MPa) [33]. This may show that the contralateral reversed LCP-DF single-plate fixation procedure has a high potential risk of implant failure under full weight-bearing [15, 26, 34]. Moreover, the slope of the load-strain curve of the single-plate method changed at a strain of 400 × 10^− 6^, due to the contact of the plate with proximal lateral side of the distal bone fragment. This revealed that the single-plate procedure offered a weaker fixation, being unable to maintain the position of the proximal and the distal bone fragment, which may increase the risk of delayed of bone union and implant failure under full weight-bearing. On the other hand, double-plate fixation showed a significant reduction in stress concentration in the lateral plates at the fracture site. Under full weight-bearing, the maximum stress level in the lateral plate was 268 MPa. The stress level in the plates fell within the fatigue threshold of titanium (ca. 598 MPa), corresponding to approximately 5 years of the normal functioning period [33, 35]. Likewise, the slope of the load-strain curve seemed to be constant when using double-plate fixation, revealing the strength of this fixation procedure. Taken together, double-plate fixation with reversed contralateral LCP-DF seems suitable for the treatment of Vancouver B1 periprosthetic femoral fractures in the early postoperative period for elderly patients. This fixation procedure will be further evaluated under different physiological boundary conditions that reflect the real loads imposed by patients’ routine activities.

The limitations in this study include the following: (1) our constructs were only tested for one type of fracture typified by a large defect, as we believe that the use of an extreme unstable model may be more valuable for delineating the usefulness of constructs and fixation methods. In addition, it is necessary to further evaluate our procedure using cadaveric models to determine its clinical and practical implications; (2) in our study, static loads were analysed by geometrically nonlinear analysis and not perturbation analysis, because the latter is a specific test for simulating the initial stage, and patients with periprosthetic femoral fractures are not expected to do intense exercise immediately after surgery. FEA and biomechanical testing were therefore performed to compare the usefulness of double-plating to that of a single-plating procedure. Our comparative study showed that double-plate fixation offered greater stability and strength with lesser stress on the defect site than that offered by single-plate fixation. However, appropriate mechanical forces and biophysical environment after surgery are necessary for the healing process of fractured bones via stimulating local cellular proliferation and tissue differentiation [36]. Therefore, it is important to promote micro-motions within the fractures and stimulate bone union. It was documented that the optimal strain value for bone fusion is 100 × 10^− 6^ or higher [37]. Our results showed that the strain value of double-plating is approximately 317 × 10^− 6^ under full weight-bearing, which is considered to be an appropriate strain value.

## Conclusions

This was the first study to report FEA and biomechanical testing results of double-plate fixation using reversed contralateral LCP-DF for Vancouver B1 periprosthetic femoral fractures. Adding an anterior narrow locking plate significantly reduced the stress concentration in the lateral plate at the fracture site. The present results showed that the double-plating method with reversed contralateral LCP-DF significantly increased the construct strength and might allow full weight-bearing from the early postoperative period. We will perform further study to evaluate the effects of the current fixation procedures on bone union/healing process to determine their usefulness in clinical practice.

## Data Availability

All data supporting our findings are contained within the manuscript.

## Abbreviations

THA: Total hip arthroplasty
LCP-DF: Locking compression-distal femoral plates
FEA: Finite element analysis
PMMA: polymethylmethacrylate
